# Supplementary material for: Nanohole-boosted electron transport between nanomaterials and bacteria as a concept for nano–bio interactions
Source: Nat Commun. 2021 Jan 21;12:493. doi: 10.1038/s41467-020-20547-9 (PMC7820612; doi:10.1038/s41467-020-20547-9)
Supplement: Supplementary file 1 — Supplementary Information [file 41467_2020_20547_MOESM1_ESM.pdf]

Supporting Information

Nanohole-boosted Electron Transport between Nanomaterials and Bacteria as a Concept for Nano-bio Interactions

Tonglei Shi[1, 2], Xuan Hou[1, 2], Shuqing Guo[1], Lei Zhang[1], Changhong Wei[1], Ting Peng[1], Xiangang Hu[1]*

[1]Key Laboratory of Pollution Processes and Environmental Criteria (Ministry of Education)/Tianjin Key Laboratory of Environmental Remediation and Pollution Control, College of Environmental Science and Engineering, Nankai University, Tianjin 300350, China.

[2]These authors contributed equally.

*Corresponding author: Xiangang Hu, email: huxiangang@nankai.edu.cn

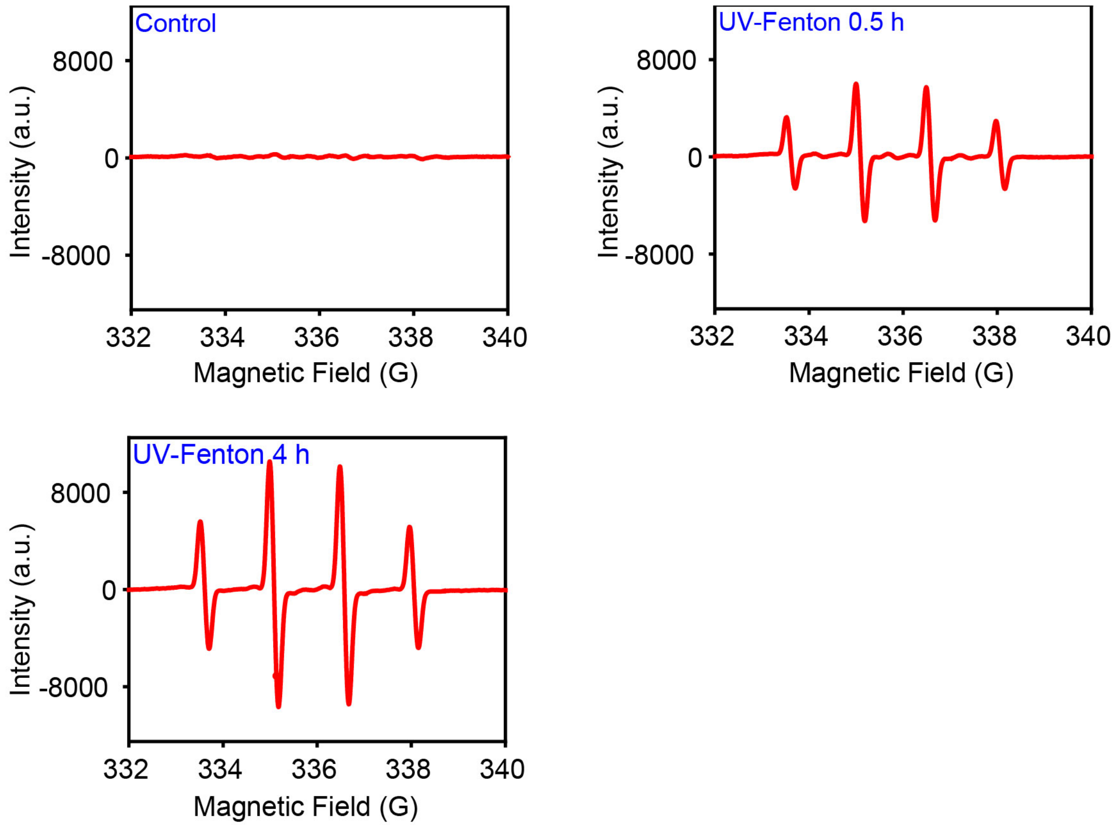

Supplementary Fig. 1. Electron spin resonance (ESR) spectra of hydroxyl radicals from the UV-Fenton reaction. ESR spectra obtained from samples containing (a) 5,5-dimethyl-1-pyrroline-N-oxide (DMPO) without the UV-Fenton reaction (control), (b) DMPO after the UV-Fenton reaction for 0.5 h and (c) DMPO after the UV-Fenton reaction for 4 h. Source data are provided as a Source Data file.

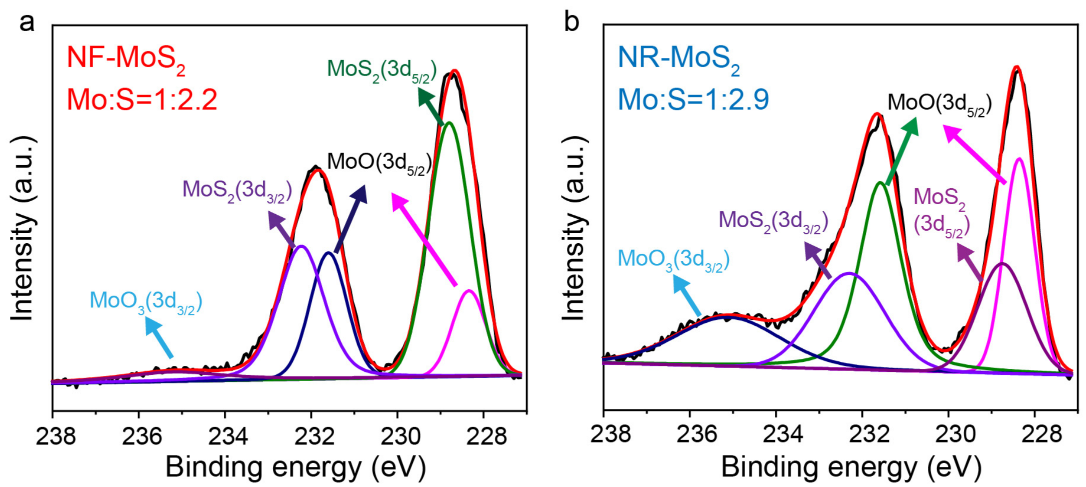

Supplementary Fig. 2. X-ray photoelectron spectroscopy (XPS) analysis of (a) NF-MoS_2 (nanohole-free MoS_2) and (b) NR-MoS_2 (nanohole-enriched MoS_2). Source data are provided as a Source Data file.

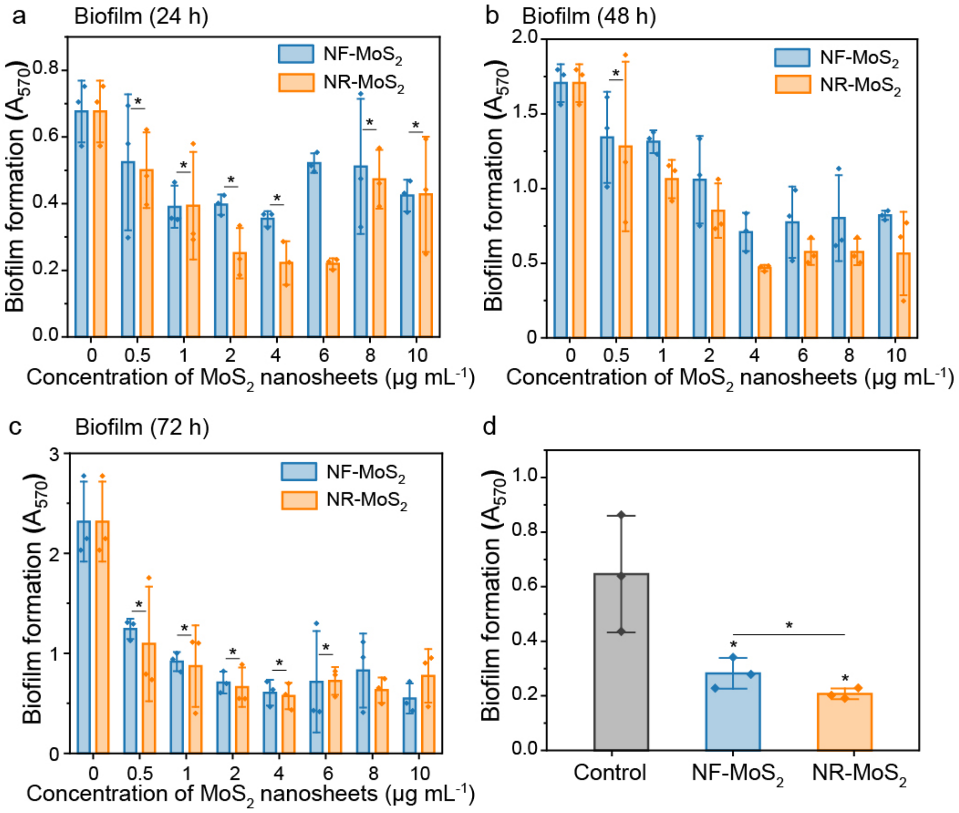

Supplementary Fig. 3. *S. aureus* biofilms were inhibited by NF-MoS_2 and NR-MoS_2 at 0, 0.5, 1.0, 2.0, 4.0, 6.0, 8.0 and 10.0 μg mL^{-1}. The biofilms were grown for (a) 24 h, (b) 48 h, and (c) 72 h. (d) Planktonic *S. aureus* cells cocultured with 4.0 μg mL^{-1} MoS_2 nanosheets for 24 h before biofilm formation. Control: Untreated *S. aureus*. Data represent the mean ± SD (n = 3 biologically independent samples). Significance was assessed using a two-sided student's t-test with multiple comparisons: *, $p < 0.05$. Source data are provided as a Source Data file.

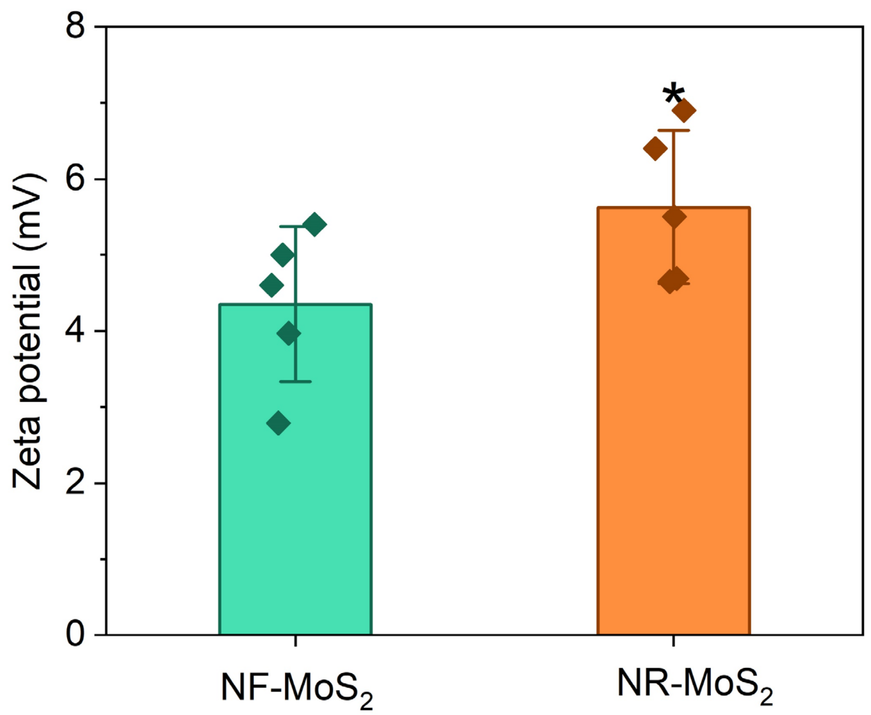

Supplementary Fig. 4. Zeta potential of NF-MoS_2 and NR-MoS_2 at 10 μg mL^{-1} in tryptic soy broth (TSB). Data represent the mean ± SD (n = 5 biologically independent samples). Significance was assessed using a two-sided student's t-test with multiple comparisons: *, $p < 0.05$. Source data are provided as a Source Data file.

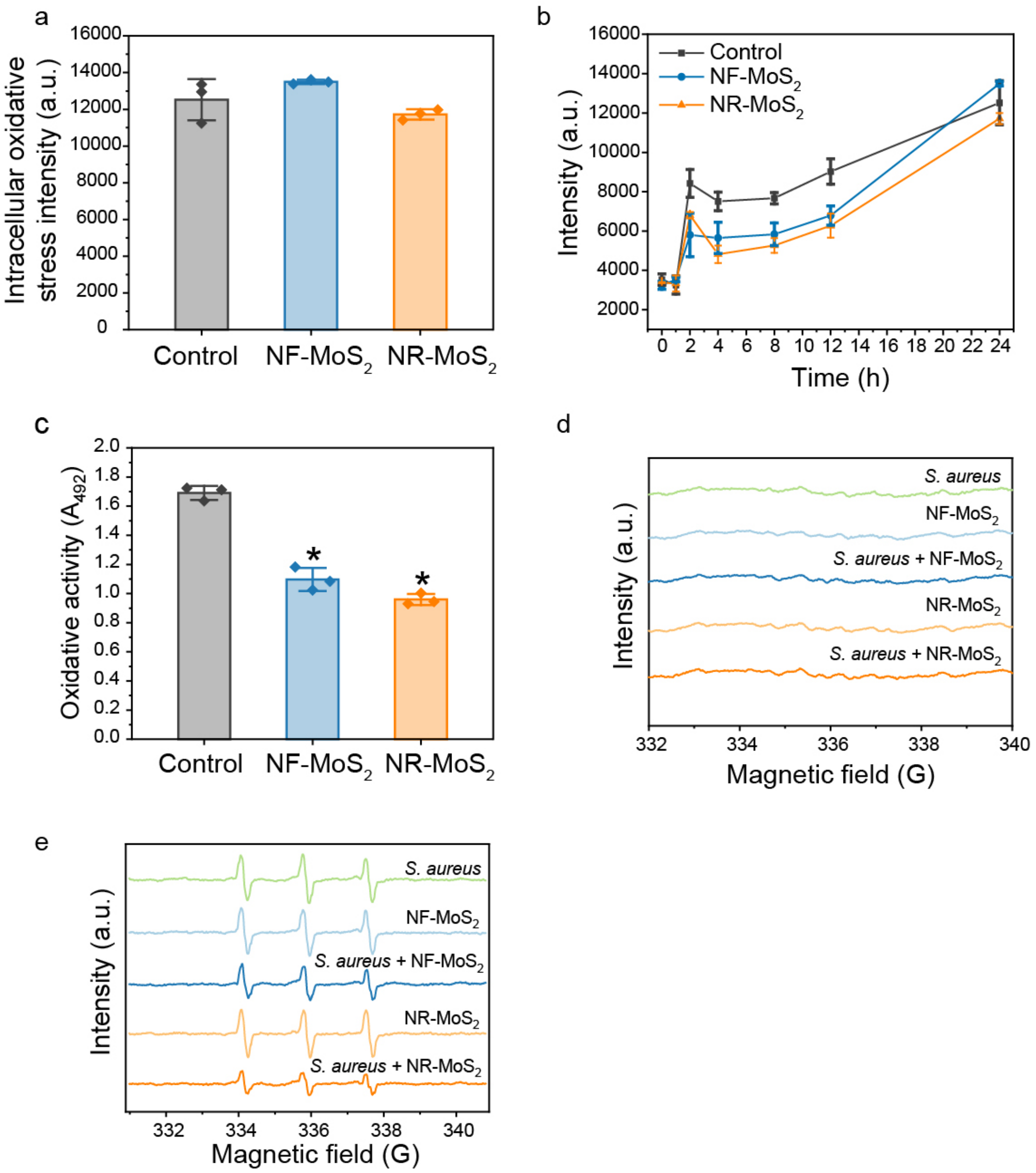

Supplementary Fig. 5. *S. aureus* cells treated with NF-MoS_2 and NR-MoS_2. (a) Intracellular oxidative stress levels of *S. aureus* cells treated with NF-MoS_2 and NR-MoS_2 at 10 μg mL^{-1}. Control: untreated *S. aureus*. (b) Time-dependent intracellular oxidative stress levels. Control: untreated *S. aureus*. (c) Analysis of biofilm oxidative activity of *S. aureus* treated with NF-MoS_2 and NR-MoS_2 at 10 μg mL^{-1} through the 2,3-Bis[2-methyloxy-4-nitro-5-sulfophenyl]-2H-tetrazolium-5-carboxanilide (XTT) assay. Control: untreated *S. aureus*. (d) ESR spectra of hydroxyl radicals (·OH) trapped by DMPO. (e) ESR spectra of singlet oxygen radicals

($^1O_2$) trapped by 2,2,6,6-tetramethyl-4-piperidine (TEMP). The concentration of MoS_2 nanosheets was 10 μg mL^{-1}. Data represent the mean ± SD (n = 3 biologically independent samples). Significance was assessed using a two-sided student's t-test with multiple comparisons: *, $p < 0.05$. Source data are provided as a Source Data file.

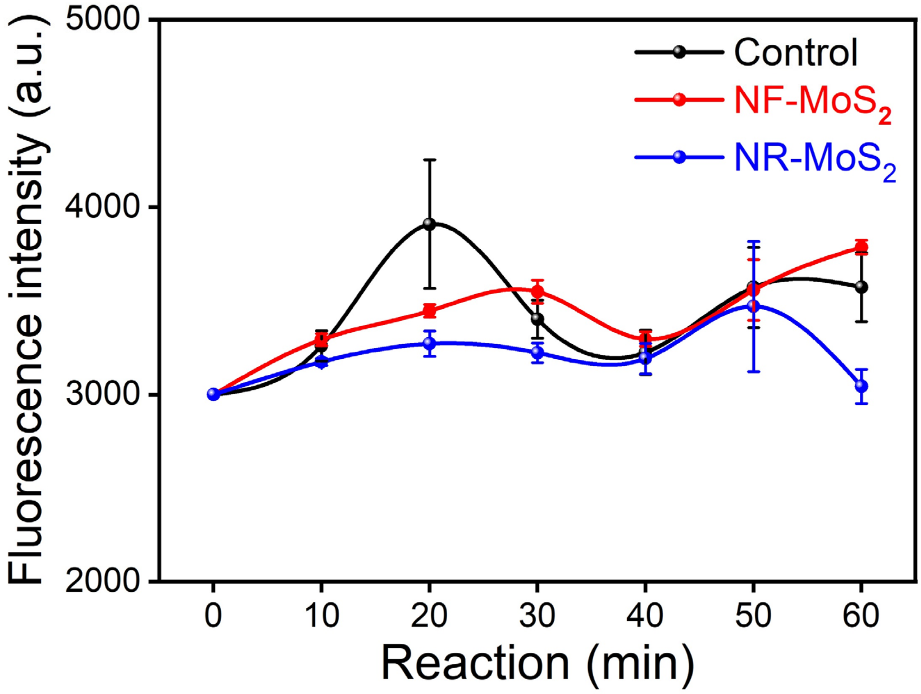

Supplementary Fig. 6. Analysis of the cell membrane potential of *S. aureus* cells treated with NF-MoS_2 and NR-MoS_2 at 10 μg mL^{-1}. Control: untreated *S. aureus*. Data represent the mean ± SD (n = 3 biologically independent samples). Source data are provided as a Source Data file.

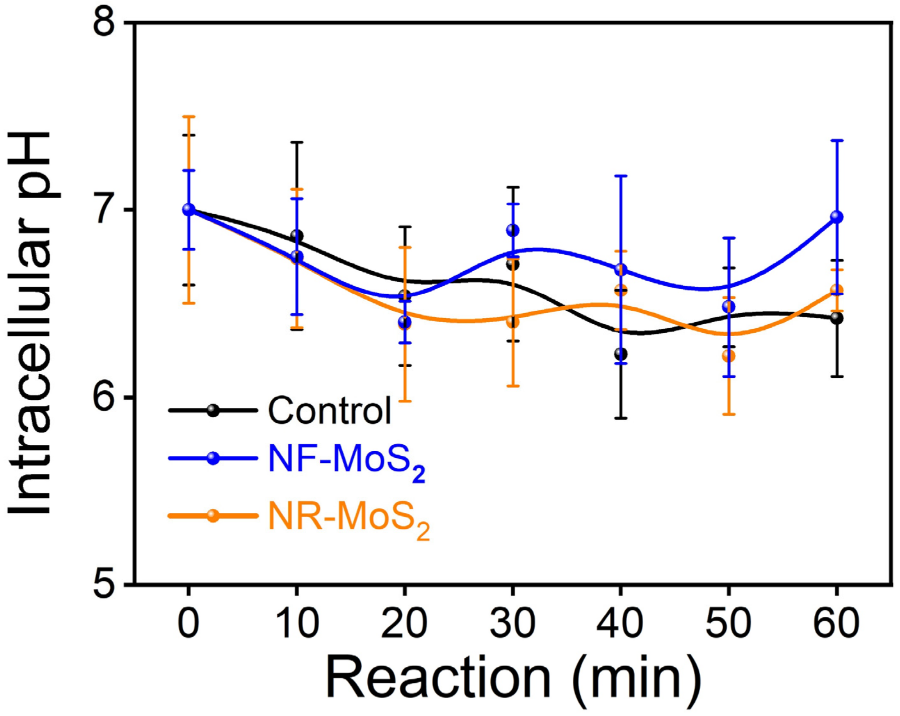

Supplementary Fig. 7. Intracellular pH changes in *S. aureus* cells induced by NF-MoS_2 and NR-MoS_2 at 10 μg mL^{-1}. Control: untreated *S. aureus*. Data represent the mean ± SD (n = 3 biologically independent samples). Source data are provided as a Source Data file.

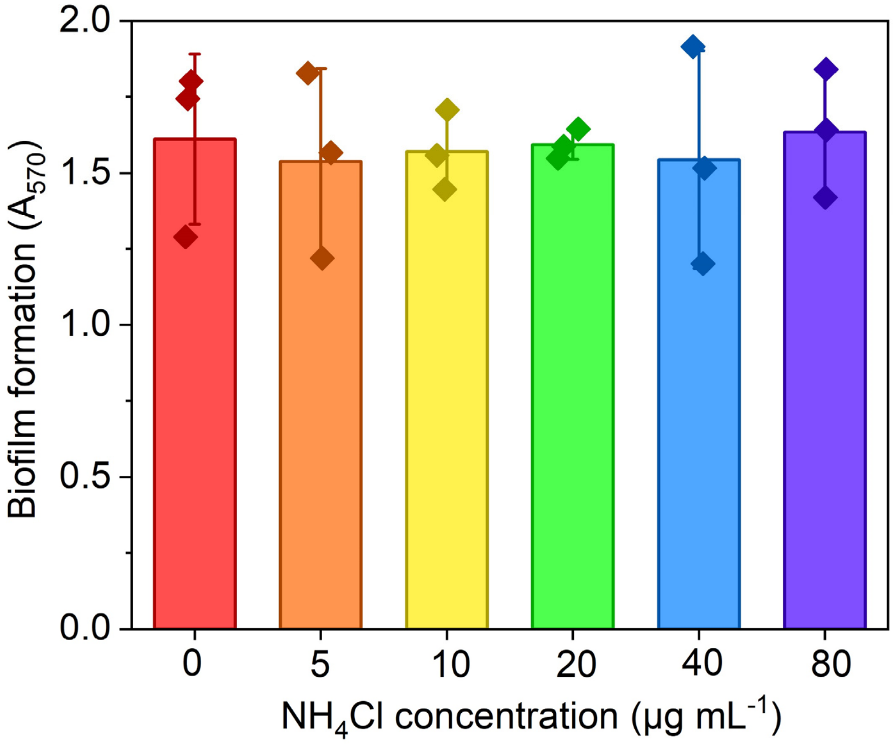

Supplementary Fig. 8. *S. aureus* biofilm treated with NH_4Cl at 5, 10, 20, 40 and 80 μg mL^{-1}. Data represent the mean ± SD (n = 3 biologically independent samples). Source data are provided as a Source Data file.

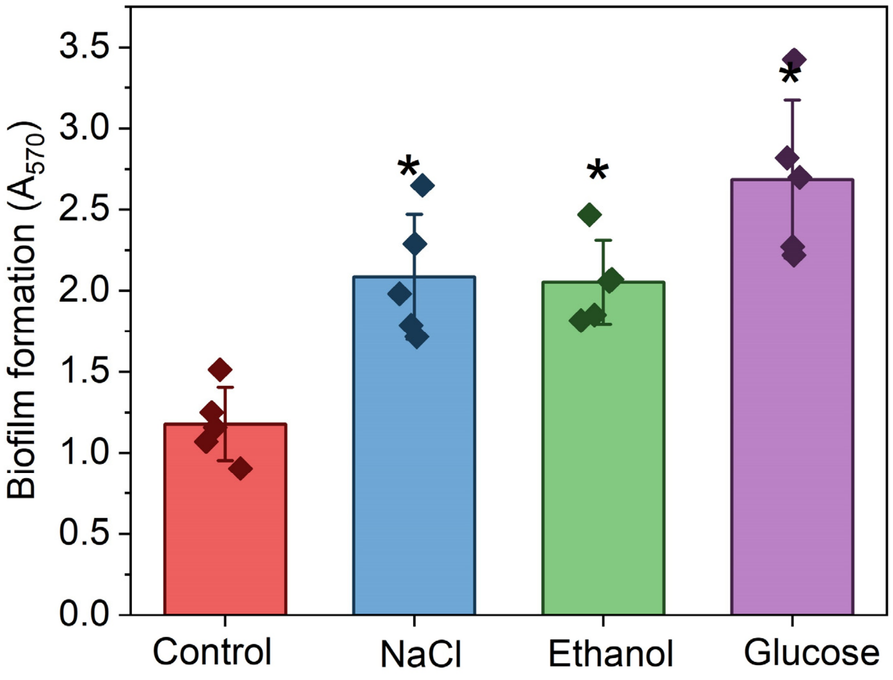

Supplementary Fig. 9. Biofilm influenced by inducers (glucose, ethanol and NaCl). The final concentrations of glucose, ethanol and NaCl in the TSB medium were 0.5%, 4% and 2%, respectively. Control: untreated *S. aureus*. Data represent the mean ± SD (n = 5 biologically independent samples). Significance was assessed using a two-sided student's t-test with multiple comparisons: *, $p < 0.05$. Source data are provided as a Source Data file.

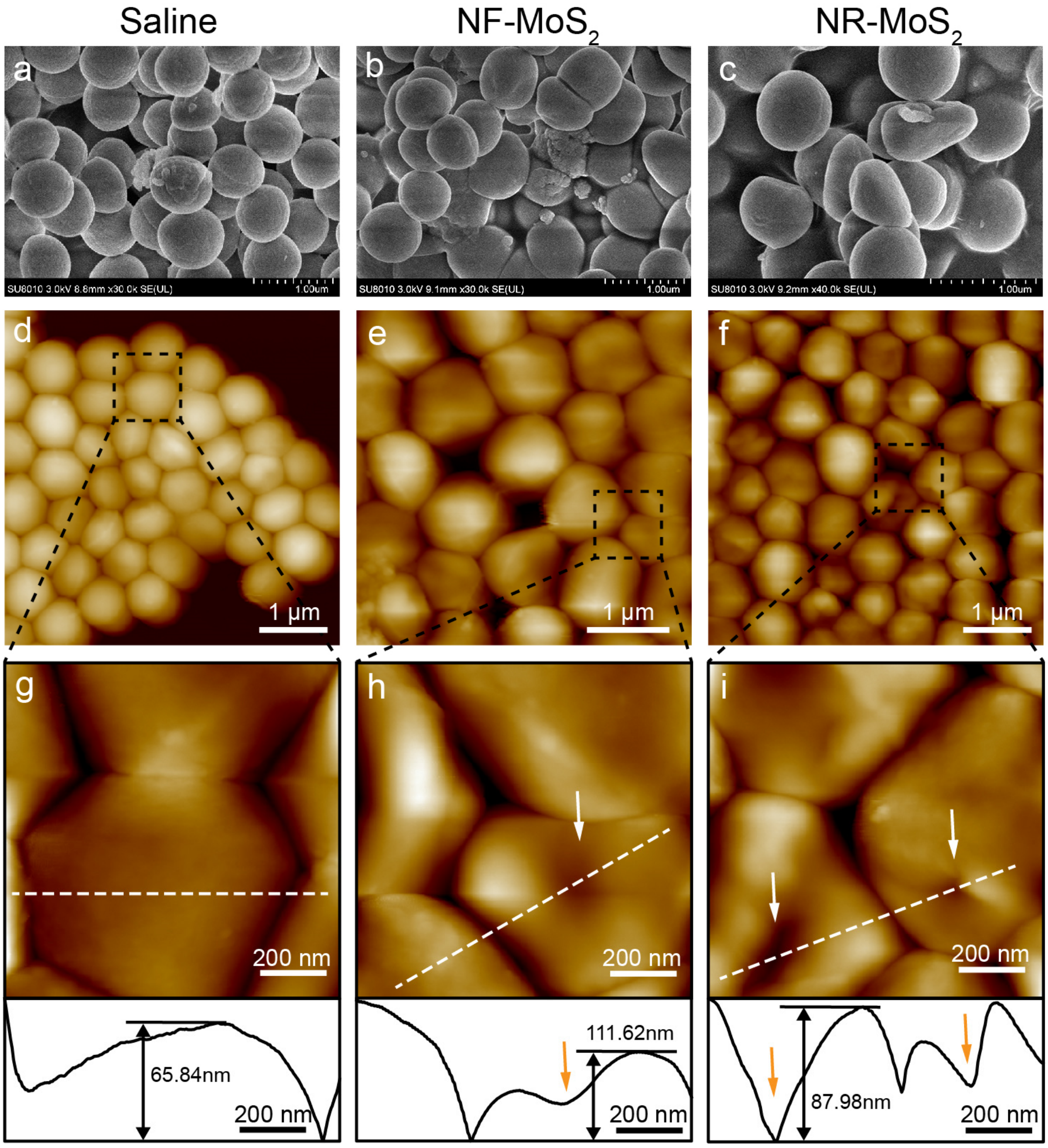

Supplementary Fig. 10. Membrane integrity and surface morphology analysis through scanning electron microscopy (SEM) and atomic force microscopy (AFM). (a)-(c) Representative SEM imaging of bacterial cells after MoS_2 nanosheet treatment. (d)-(i) Surface height topology analysis of bacterial cells after MoS_2 nanosheet treatment measured by representative AFM imaging. Concentration of MoS_2 nanosheets: 10 μg mL^{-1}. Representative micrographs were selected from three independent samples. Source data are provided as a Source Data file.

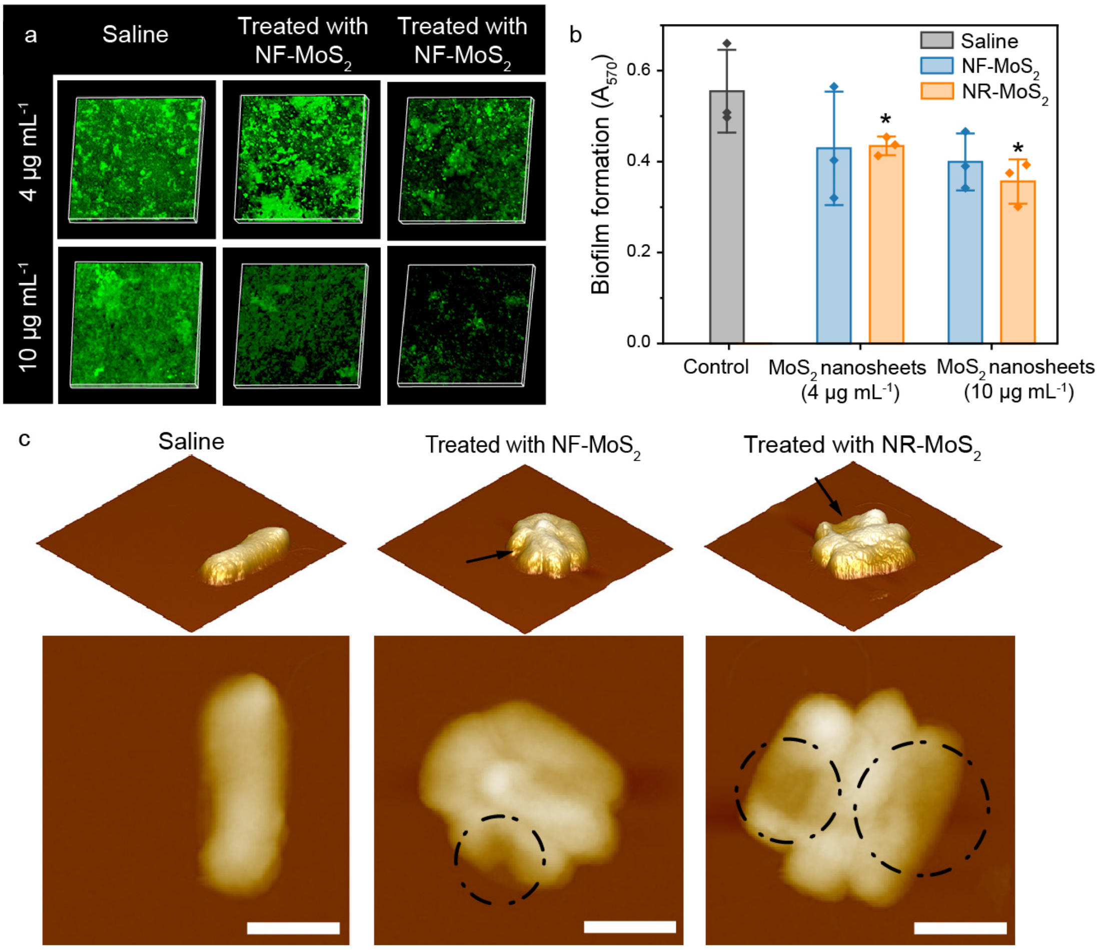

Supplementary Fig. 11. Gram-negative bacterial (*Escherichia coli*, *E. coli*) biofilm formation was influenced by NF-MoS_2 and NR-MoS_2. (a) Representative 3D-reconstructed confocal images of *E. coli* biofilms treated with MoS_2 nanosheets. The length and width were both 800 nm. (b) *E. coli* biofilm inhibited by NF-MoS_2 and NR-MoS_2 at 0, 4.0 and 10.0 μg mL^{-1}. The biofilm was grown for 24 h and then treated with MoS_2 nanosheets for 24 h. Data represent the mean ± SD (n = 3 biologically independent samples). Significance was assessed using a two-sided student's t-test with multiple comparisons: *, $p < 0.05$. (c) Surface height topology analysis of *E. coli*. Black arrows indicate hollows on the cell surface. Scale bar: 1 μm. Source data are provided as a Source Data file.

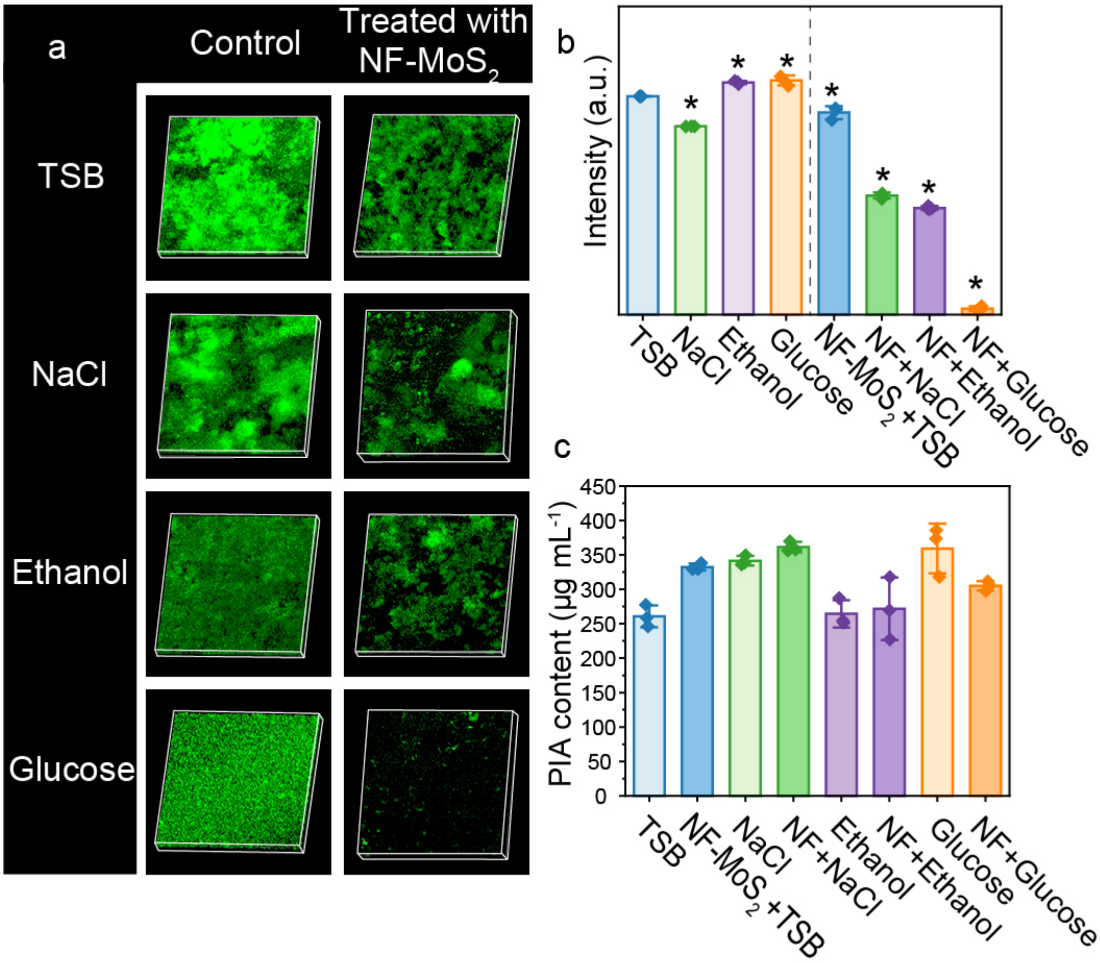

Supplementary Fig. 12. Biofilm formation influenced by NF-MoS_2 and inducers. (a) Representative 3D-reconstructed confocal images of biofilms treated with NF-MoS_2 (NF). The length and width were both 800 nm. (b) Calculated florescence intensity from 3D-reconstructed confocal images of the biofilms. The biofilms were double-stained with SYTO9 and PI. (c) Polysaccharide intercellular adhesin (PIA) contents of the biofilms. The biofilm inducers glucose (G), ethanol (E) and NaCl (N) were added to the TSB medium to final concentrations of 0.5%, 4% and 2%, respectively. The concentration of NF-MoS_2 was 2 μg mL^{-1}. Data represent the mean ± SD (n = 3 biologically independent samples). Significance was assessed using a two-sided student's t-test with multiple comparisons: *, $p < 0.05$. Source data are provided as a Source Data file.

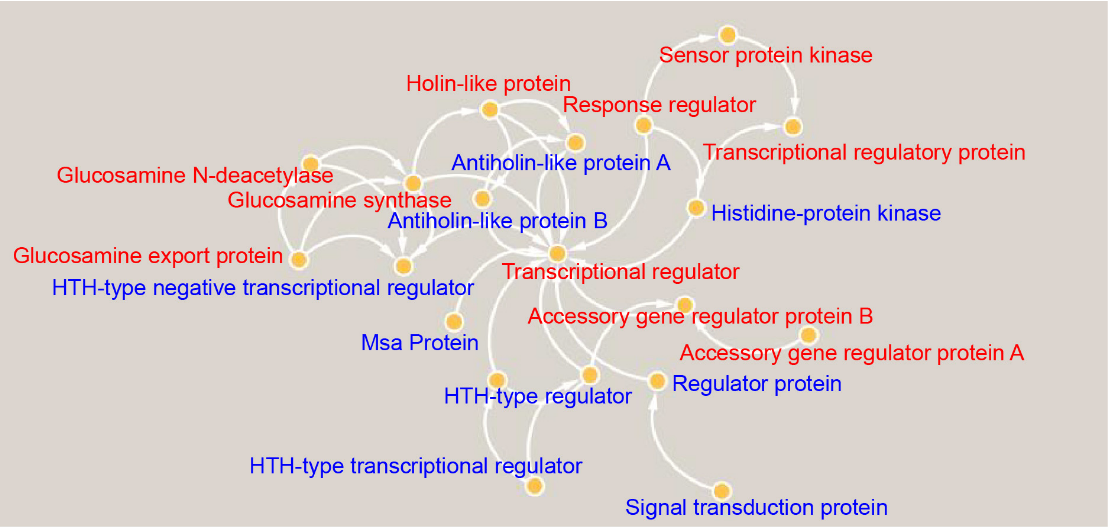

Supplementary Fig. 13. Protein-protein interaction analysis of the biofilm-related proteins of *S. aureus* after NR-MoS_2 treatment. The red and blue fonts represent down- and upregulated proteins, respectively.

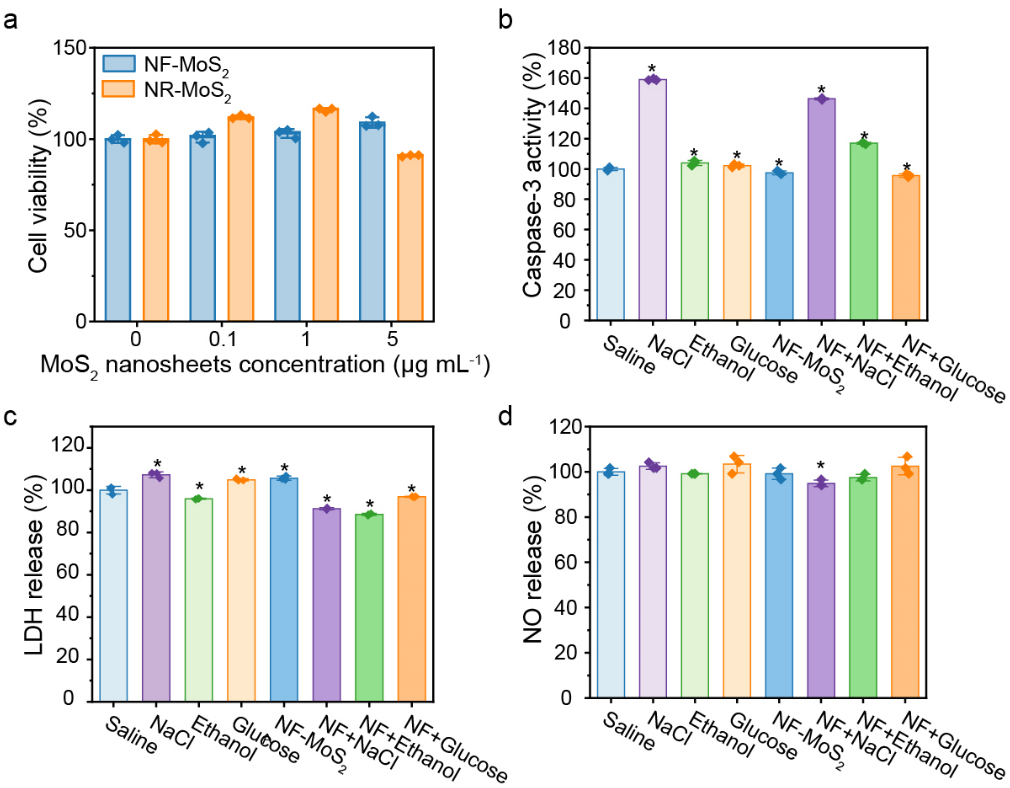

Supplementary Fig. 14. NF-MoS_2 inhibits *S. aureus* adhesion to and invasion of human cells. (a) Caco-2 cell viability after incubation with NF-MoS_2 and NR-MoS_2 at 0.1, 1.0 and 5.0 μg mL^{-1}. (b) Caspase-3 activity. (c) Lactate dehydrogenase (LDH) release. (d) NO release. The biofilm inducers glucose (G), ethanol (E) and NaCl (N) were added to the TSB medium to final concentrations of 0.5%, 4% and 2%, respectively. The concentration of NF-MoS_2 was 2 μg mL^{-1}. Data represent the mean ± SD (n = 3 biologically independent samples). Significance was assessed using a two-sided student's t-test with multiple comparisons: *, $p < 0.05$. Source data are provided as a Source Data file.

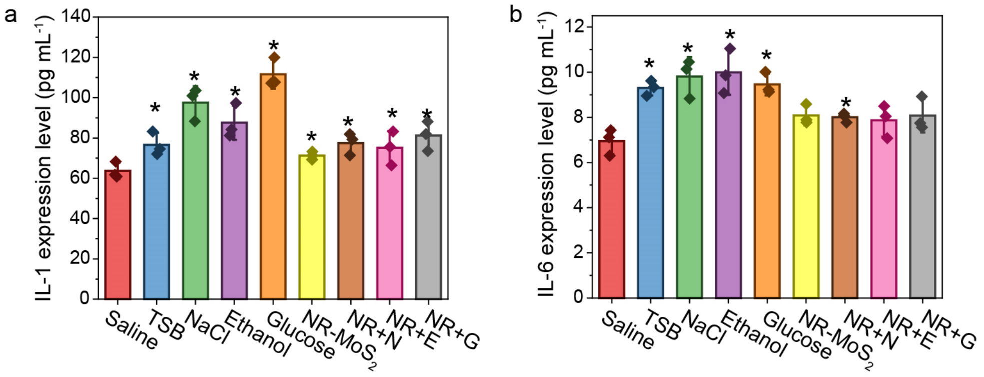

Supplementary Fig. 15. Cytokine (a) IL-1 and (b) IL-6 expression. The biofilm inducers glucose (G), ethanol (E) and NaCl (N) were added to the TSB medium to final concentrations of 0.5%, 4% and 2%, respectively. *S. aureus* (10^8 CFU mL^{-1}, 100 μL) and NR-MoS_2 (4 μg mL^{-1}, 100 μL) were injected into ICR mice through the tail vein. Data represent the mean ± SD (n = 3 biologically independent samples). Significance was assessed using a two-sided student's t-test with multiple comparisons: *, $p < 0.05$. Source data are provided as a Source Data file.

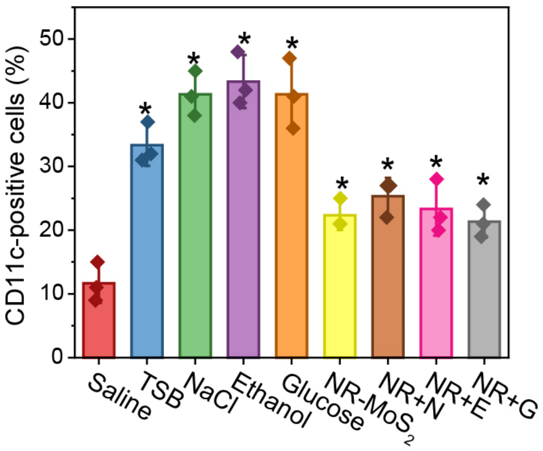

Supplementary Fig. 16. Percentage of CD11c-positive cells counted by immunohistochemical analysis. The biofilm inducers glucose (G), ethanol (E) and NaCl (N) were added to the TSB medium to final concentrations of 0.5%, 4% and 2%, respectively. *S. aureus* (10^8 CFU mL^{-1}, 100 μL) and NR-MoS_2 (4 μg mL^{-1}, 100 μL) were injected into ICR mice through the tail vein. Data represent the mean ± SD (n = 3 biologically independent samples). Significance was assessed using a two-sided student's t-test with multiple comparisons: *, $p <$ 0.05. Source data are provided as a Source Data file.

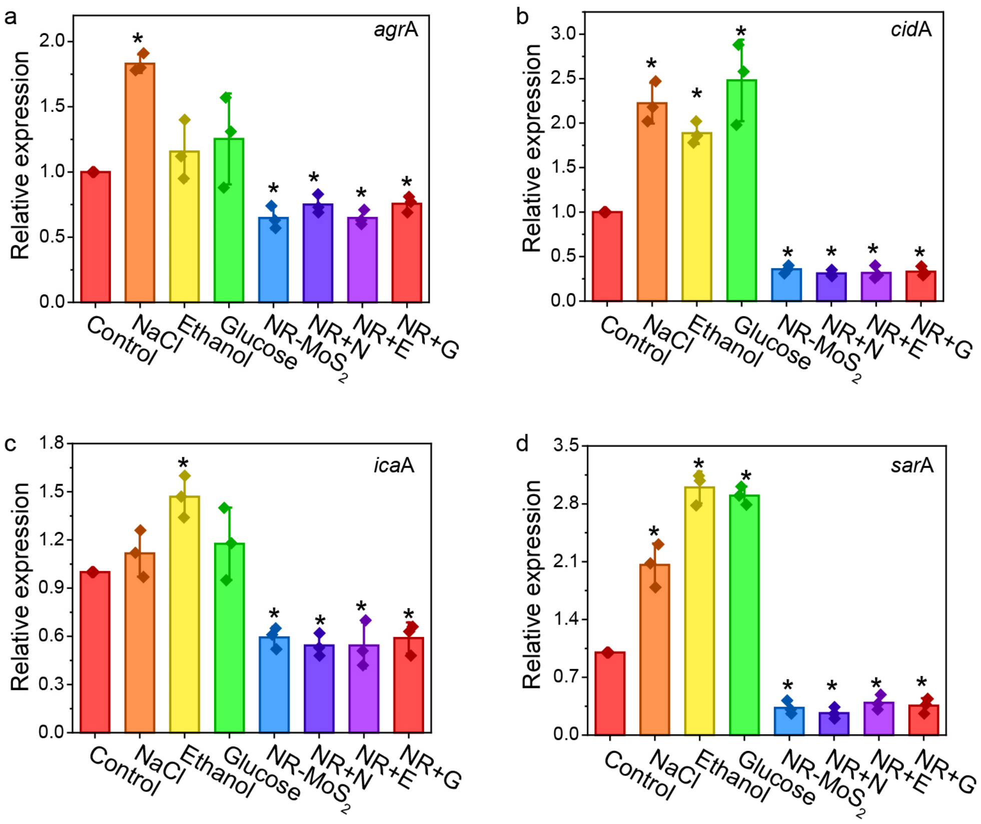

Supplementary Fig. 17. Relative expression of biofilm-related genes quantified by real-time PCR. Relative expression of (a) *agr*A, (b) *cid*A, (c) *ica*A, (d) *sar*A genes. The biofilm inducers glucose (G), ethanol (E) and NaCl (N) were added to the TSB medium to final concentrations of 0.5%, 4% and 2%, respectively. The concentration of NR-MoS_2 was 2.0 μg mL^{-1}. Control: untreated *S. aureus*. Data represent the mean ± SD (n = 3 biologically independent samples). Significance was assessed using a two-sided student's t-test with multiple comparisons: *, p < 0.05. Source data are provided as a Source Data file.

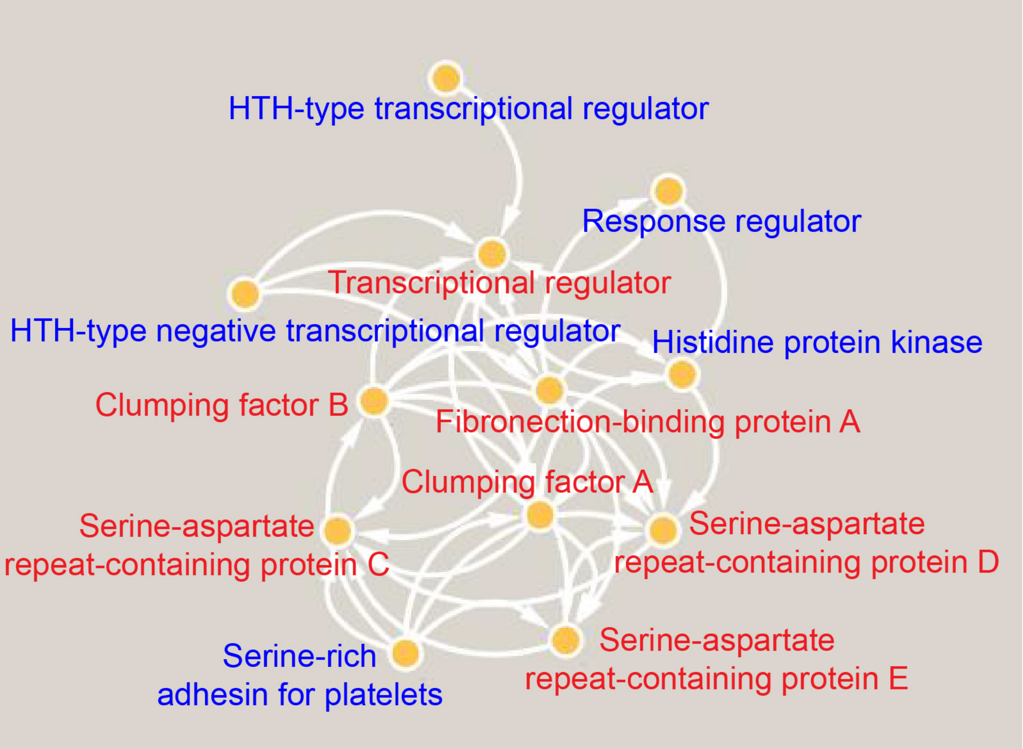

Supplementary Fig. 18. Protein-protein interaction analysis of the adhesion- and invasion-related proteins of *S. aureus* after NR-MoS_2 treatment. The red and blue fonts represent down- and upregulated proteins, respectively.

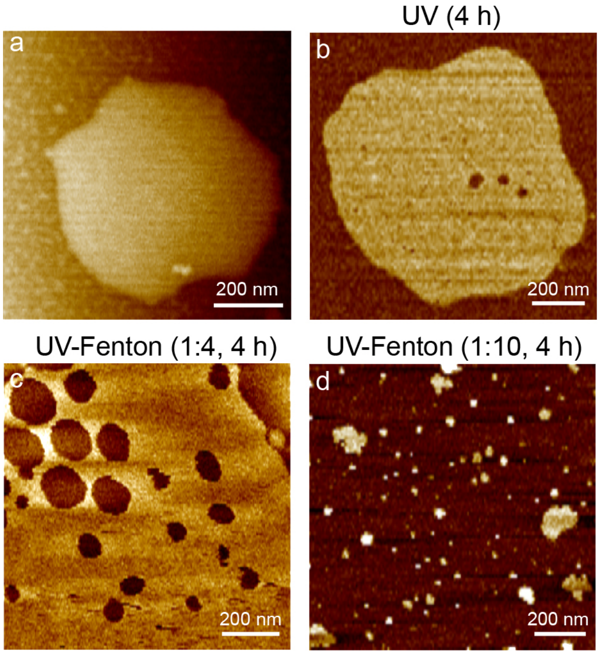

Supplementary Fig. 19. Representative AFM observations of NR-MoS_2 nanosheets prepared using different reaction conditions. (a) Pristine MoS_2 (NF-MoS_2) nanosheets. (b) NF-MoS_2 treated with UV lamp irradiation for 4 h. (c) NF-MoS_2 treated with $FeSO_4$ and H_2O_2 at a volume ratio of 1:4 under UV lamp irradiation for 4 h. (d) NF-MoS_2 treated with $FeSO_4$ and H_2O_2 at a volume ratio of 1:10 under UV lamp irradiation for 4 h. UV lamp: 150 W; $FeSO_4$ solution: 1 mM; H_2O_2 solution: 50 mM. Representative micrographs were selected from three independent samples.

Supplementary Table 1. Genes and primer sequences used for quantitative real-time polymerase chain reaction (PCR).

Genes	Primers (5'-3')
*ica*A	F: GATACTGATATGATTACCGAAGAT
	R: GAACCAACATCCAACACAT
*cid*A	F: ATTCATAAGCGTCTACACCTT
	R: TTCTTCATACCGTCAGTTGT
*agr*A	F: TGAAATTCGTAAGCATGACCC
	R: CATCGCTGCAACTTTGTAGAC
*sar*A	F: TGTTTGCTTCAGTGATTCGTTTA
	R: AACCACAAGTTGTTAAAGCAGTTA
16S rRNA	F: CGTGCTACAATGGACAATACAAA
	R: ATCTACGATTACTAGCGATTCCA
