# Supplementary material for: Nanohole-boosted electron transport between nanomaterials and bacteria as a concept for nano–bio interactions
Source: Nat Commun. 2021 Jan 21;12:493. doi: 10.1038/s41467-020-20547-9 (PMC7820612; doi:10.1038/s41467-020-20547-9)
Supplement: Supplementary file 3 — Reporting Summary [file 41467_2020_20547_MOESM3_ESM.pdf]

nature research

Corresponding author(s): Xiangang Hu

Last updated by author(s): Nov 17, 2020

Reporting Summary

Nature Research wishes to improve the reproducibility of the work that we publish. This form provides structure for consistency and transparency in reporting. For further information on Nature Research policies, see our Editorial Policies and the Editorial Policy Checklist.

Statistics

For all statistical analyses, confirm that the following items are present in the figure legend, table legend, main text, or Methods section.

n/a	Confirmed	
☐	☒	The exact sample size (*n*) for each experimental group/condition, given as a discrete number and unit of measurement
☐	☒	A statement on whether measurements were taken from distinct samples or whether the same sample was measured repeatedly
☐	☒	The statistical test(s) used AND whether they are one- or two-sided<br>*Only common tests should be described solely by name; describe more complex techniques in the Methods section.*
☐	☒	A description of all covariates tested
☒	☐	A description of any assumptions or corrections, such as tests of normality and adjustment for multiple comparisons
☐	☒	A full description of the statistical parameters including central tendency (e.g. means) or other basic estimates (e.g. regression coefficient) AND variation (e.g. standard deviation) or associated estimates of uncertainty (e.g. confidence intervals)
☐	☒	For null hypothesis testing, the test statistic (e.g. *F*, *t*, *r*) with confidence intervals, effect sizes, degrees of freedom and *P* value noted<br>*Give P values as exact values whenever suitable.*
☒	☐	For Bayesian analysis, information on the choice of priors and Markov chain Monte Carlo settings
☒	☐	For hierarchical and complex designs, identification of the appropriate level for tests and full reporting of outcomes
☐	☒	Estimates of effect sizes (e.g. Cohen's *d*, Pearson's *r*), indicating how they were calculated

Our web collection on statistics for biologists contains articles on many of the points above.

Software and code

Policy information about availability of computer code

Data collection	No software was used.
Data analysis	All the all-atom molecular dynamics were based on a general AMBER force field with the RESP charges and performed using the Gromacs-4.6.7 software package. Statistical analysis were analyzed using GraphPad Prism software (GraphPad, USA). The details are provided in the method section.

For manuscripts utilizing custom algorithms or software that are central to the research but not yet described in published literature, software must be made available to editors and reviewers. We strongly encourage code deposition in a community repository (e.g. GitHub). See the Nature Research guidelines for submitting code & software for further information.

Data

Policy information about availability of data

All manuscripts must include a data availability statement. This statement should provide the following information, where applicable:

- Accession codes, unique identifiers, or web links for publicly available datasets
- A list of figures that have associated raw data
- A description of any restrictions on data availability

A list of figures that have associated raw data.

Field-specific reporting

Please select the one below that is the best fit for your research. If you are not sure, read the appropriate sections before making your selection.

- [x] Life sciences
- [ ] Behavioural & social sciences
- [ ] Ecological, evolutionary & environmental sciences

For a reference copy of the document with all sections, see nature.com/documents/nr-reporting-summary-flat.pdf

Life sciences study design

All studies must disclose on these points even when the disclosure is negative.

Sample size	All the experiments were performed in triplicate and in parallel, and eight replicates of the animal experiments were performed in parallel. All the data were analyzed using GraphPad Prism software (GraphPad, USA) and are presented as the means and standard errors. Comparisons between different groups were performed with Student's t-test. For all tests, $p < 0.05$ was considered to indicate statistical significance. These sample sizes are sufficient to control errors produced by biological experiments .
Data exclusions	No data were excluded from the analyses.
Replication	All the experiments were performed in triplicate and in parallel, and eight replicates of the animal experiments were performed in parallel. All attempts at replication were successful.
Randomization	To ensure the scientific difference between the experimental groups and control groups, the samples that have the similar properties (e.g., the age and size of rats) to the control, were randomly allocated into experimental groups.
Blinding	Blinding was not relevant to our study, because the tested samples are cells, rats and mouse.

Reporting for specific materials, systems and methods

We require information from authors about some types of materials, experimental systems and methods used in many studies. Here, indicate whether each material, system or method listed is relevant to your study. If you are not sure if a list item applies to your research, read the appropriate section before selecting a response.

Materials & experimental systems

n/a	Involved in the study	
x		Antibodies
	x	Eukaryotic cell lines
x		Palaeontology and archaeology
	x	Animals and other organisms
x		Human research participants
x		Clinical data
x		Dual use research of concern

Methods

n/a	Involved in the study	
x		ChIP-seq
x		Flow cytometry
x		MRI-based neuroimaging

Eukaryotic cell lines

Policy information about cell lines

Cell line source(s)	Human intestinal adenocarcinoma cells (Caco-2 cells)
Authentication	Human intestinal adenocarcinoma cells (Caco-2 cells), which were obtained from Dingguo Changsheng Biotech Co., Ltd. (Beijing, China).
Mycoplasma contamination	All cell lines tested negative for Mycoplasma contamination.
Commonly misidentified lines (See ICLAC register)	None misidentified lines were used.

Animals and other organisms

Policy information about studies involving animals; ARRIVE guidelines recommended for reporting animal research

Laboratory animals	Healthy 4-week ICR mice (SPF) and Sprague-Dawley (SD) rats.
Wild animals	The study did not involve wild animals.

Field-collected samples	The study did not involve samples collected from the field.
Ethics oversight	All the experiments were performed according to the Guidelines established by the Animal Care and Use Committee of Nankai University (Tianjin, China).

Note that full information on the approval of the study protocol must also be provided in the manuscript.
